# Experiences of peer victimization and teacher support in secondary school predict university enrolment 5 years later: Role of school engagement

**DOI:** 10.1111/bjep.12500

**Published:** 2022-03-25

**Authors:** Eva Grew, Gülseli Baysu, Rhiannon N. Turner

**Affiliations:** ^1^ Queen’s University Belfast Belfast UK

**Keywords:** aspirations, minority, peer victimization, school engagement, teacher support, university

## Abstract

**Background:**

Peer victimization has an adverse effect on academic outcomes. However, longitudinal research on how peer victimization affects access to higher education is lacking.

**Aims:**

In this study, we investigated the mechanisms through which peer victimization and teacher support affect aspirations for and enrolment at university 5 years later through engagement in secondary school. We also examined whether these effects were moderated by ethnicity, and whether teacher support may compensate for the effects of peer victimization.

**Sample:**

The sample (*N* = 15,110, 51% male, 68% White, 12% Black and 20% Asian) was drawn from a nationally representative study of young people in England. We used data from four waves, following adolescents over 3 years of secondary education (T1–T2–T3, age 13 to 15 years) until university (T4, age 18 years).

**Method:**

Data were analysed in a longitudinal structural equation model in Mplus 8.

**Results:**

Adolescents subjected to more peer victimization at T1 had lower university aspirations 2 years later and were less likely to attend university 5 years later. These effects were mediated via secondary school engagement. Teacher support at T1 was related to higher school engagement, leading to higher aspirations (T3) and higher likelihood of university enrolment (T4) over time. We also found evidence that teacher support may lessen the effect of peer victimization on school engagement, and that ethnic background may moderate the effect of teacher support.

**Conclusions:**

Peer victimization had a small long‐lasting negative effect on university choices via school engagement, while teacher support had a positive effect. In summary, relationships in secondary school have long‐lasting implications for university aspirations and enrolment.

## INTRODUCTION

Around half of young people in England enter university (Department for Education [DfE], 2019) and most of them do so within a few years of completing their secondary education. The decision to attend university must therefore be established sometime before finishing secondary education. In other words, aspirations developed during secondary school are important for the path to university (Christofides et al., 2015). Promoting positive university aspirations is vital in terms of widening access to universities, as fostering aspirations in secondary school may encourage pupils’ later enrolment at university (Klasik, 2012). However, the efforts should not simply focus on raising aspirations, as these are already high among young people (Rainford, 2021). Most adolescents, regardless of their ethnic group, aspire to attend university (Berrington et al., 2016; Schneider & Saw, 2016). Instead, the research should explore whether university aspirations translate into actual university enrolment, as in the above‐cited US‐based study by Schneider and Saw (2016), adolescents in certain ethnic groups (viz. Black, Hispanic and multiracial) had lower predicted rates of enrolment than others, despite their high aspirations. Contrarily, in the United Kingdom, the White ethnic group had the lowest educational aspirations (Berrington et al., 2016) as well as the lowest enrolment rates in higher education (The Universities & Colleges Admissions Service [UCAS], 2020). Therefore, aspirations may not translate into university attendance equally in all ethnic groups. However, longitudinal research on how socio‐ecological circumstances around adolescents influence their path to university is scarce. In the present study, we focused on ethnic minority and majority adolescents’ university aspirations and enrolment.

**FIGURE 1:**
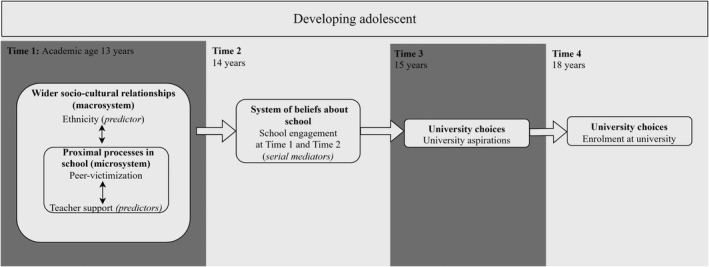
Predicted model describing a path to university influenced by social experiences at time 1

Since secondary school relationships play a key role in forming aspirations (Cunninghame et al., 2020; Vernon et al., 2019), in this study, we consider secondary school relationships as crucial antecedents of adolescent development. We use the ecological systems theory developed by Bronfenbrenner (1977) to explain how school relationships as the proximal processes in adolescents’ micro‐system can influence their developmental outcomes. Perna (2006) further applies the ecological systems theory to explain how adolescents’ university choices (aspirations and enrolment) are shaped by their internalized beliefs (embodied in habitus), the immediate social contexts like school and the wider societal context. Using the ecological systems approach to university choices (Bronfenbrenner, 1977; Perna, 2006), we propose the theoretical model in Figure 1. Accordingly, adolescents’ individual relationships in school (micro‐system) and their ethnic‐racial background (macro‐system) should additively and interactively predict their beliefs about school (reflected by their engagement in school) and in turn their academic aspirations and university enrolment as developmental outcomes. We therefore propose that social relationships in early secondary school years affect adolescents’ university choices over several years. We specifically focus on peer and teacher relationships, as relationships outside of a home context gain importance during adolescence (Eccles & Roeser, 2011), including both negative and positive school relationships; we namely focus on adolescents’ individual experiences of peer victimization and teacher support.

### Peer victimization

Negative peer relationships are a part of the school micro‐system (Bronfenbrenner, 1977), and, sadly, a large proportion of adolescents encounter peer victimization in school. According to United Nations International Children's Emergency Fund (2018), half of the world's teens face peer violence in school and peer victimization is related to poor school outcomes (Espelage et al., 2013; Fry et al., 2018; Nakamoto & Schwartz, 2010). Few studies have researched the longitudinal association between peer victimization and school outcomes. Buhs (2005) found that in children, early peer rejection was associated with later peer exclusion and peer victimization. In turn, both peer exclusion and victimization predicted lower classroom engagement. Similarly, in a short‐term longitudinal study by Schwartz et al. (2005), peer victimization was associated with worse academic functioning. Both studies were, however, investigating the longitudinal effects of victimization in children over a limited period and on other academic outcomes than university aspirations and enrolment. In a recent meta‐analysis by Polanin et al. (2021), the authors did not find a statistically significant longitudinal association between school peer victimization and school outcomes, although only seven longitudinal studies (of the 114 included in this study) researched specifically the association between being peer victimized and school outcomes. Thus, longitudinal research in this area is scarce and to our knowledge, no study has explored the longitudinal association between peer victimization and university enrolment.

According to ecological systems theory (Bronfenbrenner, 1977), development must consider environmental influences beyond the immediate situation, as environmental structure and social relationships interact. Therefore, we believe that ethnicity (as a part of the macro‐system) interacts with school relationships. For instance, students from ethnic minority groups are often at higher risk of experiencing peer victimization (Hong & Espelage, 2012), and some findings suggest that they may also be particularly susceptible to negative outcomes of victimization experiences, although the evidence is not clear. Thijs and Verkuyten (2008) found that the detrimental impact of peer victimization on academic achievement was comparable for the ethnic majority and minority adolescents, whereas Peguero (2011) found that victimized minority students were at higher risk of drop‐out. More research is needed to explore this question.

### Student–teacher relationship

In line with ecological systems theory (Bronfenbrenner, 1977), student–teacher relationships help to shape the social ecology of adolescents’ environment (Bouchard & Smith, 2017). Accordingly, previous studies consistently show that teachers play a central role in adolescents’ academic adjustment (McGrath & Van Bergen, 2015). In a meta‐analysis of over 200 studies, teacher support was significantly associated with higher school engagement and achievement both concurrently and longitudinally (Roorda et al., 2017), and the association between teacher support and achievement is even stronger for ethnic minority students (Roorda et al., 2011), suggesting that minority pupils may benefit more from supportive student–teacher relationships (Sabol & Pianta, 2012). Yet, given the inconsistent findings about university aspirations and access among different ethnic groups (Berrington et al., 2016; Schneider & Saw, 2016), it is unclear which ethnic group (if any) could benefit the most from teacher support.

In their review, Sabol and Pianta (2012) argue that supportive student–teacher relationships may moderate the developmental trajectories of at‐risk children, including children with demographic risks (such as minority ethnicity discussed above) and adjustment or academic problems; and one of the risk factors for adolescents’ adjustment and academic outcomes is peer victimization (Espelage et al., 2013). Few studies have explored the interactive effect of teacher support and peer victimization in schools, providing some evidence to the idea that a positive student–teacher relationship compensates for the negative impact of peer victimization on academic outcomes, such as perceived safety in the classroom (Boulton et al., 2009) or school engagement (Bedeck, 2015). Crucially, however, supportive student–teacher relationship also provides a much‐needed positive social experience to help adolescents overcome the troubled relationships with their peers, by helping them develop autonomy and belonging in school (McGrath & Van Bergen, 2015). Accordingly, research on the protective and risk factors in development posits that positive relationships (with peers or teachers) can protect adolescents against various stressors, including peer victimization (Espelage et al., 2013; McGrath & Van Bergen, 2015), although there are mixed findings. For example, Galand and Hospel (2013) have not found a significant interaction between teacher support and peer victimization on school disaffection, suggesting that while teacher support is a positive resource for adolescents, it may not compensate for the negative peer relationships in school. Considering the limited evidence and lack of longitudinal studies, the potential buffering role of teacher support against peer victimization is worth exploring.

### The mediating role of school engagement

In our proposed model, we suggest that school social relationships convey into university choices through adolescents’ beliefs about secondary school. Nevertheless, research into the specifics of how peer victimization and teacher support affect later university choices is scarce, despite the extensive evidence of their impact on various other indicators of school adjustment (McGrath & Van Bergen, 2015; Nakamoto & Schwartz, 2010; Roorda et al., 2017; Sabol & Pianta, 2012). Both positive and negative relationships at the level of school micro‐system shape adolescents’ academic attitudes; therefore, the more positive social relationships adolescents have within school, the more favourably they will perceive school overall. How students actively participate in school in general (i.e., not specific to any subject), how they feel about school and their commitment to education can be summarized by the term ‘school engagement’ (Li, 2011). Studies investigating the positive role of school engagement for achievement and against school drop‐out (Fredricks et al., 2004) show that engagement is key to school success. In our present study, we therefore propose school engagement as the link connecting adolescents’ positive and negative secondary school relationships to their university choices (aspirations and enrolment).

This theoretical model (school engagement as the link between peer and teacher relationships to academic outcomes) is also supported by a wealth of empirical evidence. Findings from both cross‐sectional and longitudinal studies show that in children, negative peer influence, such as victimization and exclusion, affects their achievement through reduced engagement (Buhs, 2005; Roopa et al., 2010; Nakamoto & Schwartz, 2011). Thus, over time, peer victimization leads to lower school engagement, and in turn lower achievement. It is less clear if these results are transferable to adolescence since engagement tends to decrease with age (Li et al., 2011; Wang & Eccles, 2012). However, in a cross‐sectional study with adolescents (Zimmer‐Gembeck et al., 2006), peer relationships predicted achievement through engagement, providing support for the theory that engagement links adolescent peer relationships with their academic outcomes. Similarly, the positive association between student–teacher relationship and achievement is also mediated via school engagement (Roorda et al., 2017; Zimmer‐Gembeck et al., 2006), and the association between positive student–teacher relationships and engagement is stronger in secondary than in primary school (Roorda et al., 2017). Overall, these findings suggest that the peer and teacher relationships that adolescents experience in secondary school are connected to university choices via school engagement.

### Present study

In the present study, we aimed to answer whether (and how) secondary school relationships influence adolescents’ university choices such as aspirations (over 2 years) and enrolment (over 5 years). Including both positive and negative relationships and their interactions, we focused on adolescents’ experiences of being subjected to peer victimization and having supportive teacher relationships in secondary school. While previous research on teacher support provides clear evidence of its positive and long‐lasting effect on adolescents’ school outcomes, the longitudinal role of peer victimization in academic choices is under‐researched, with inconsistent findings. Similarly, it is unclear whether teacher support can compensate for the negative effects of peer victimization. Furthermore, the previous research on longitudinal effects of school relationships has mainly focused on other school outcomes, such as grades, rather than university choices. Feeling supported in school is associated with higher engagement, and in turn with higher university aspirations (Cunninghame et al., 2020). Similarly, feeling encouraged by teachers is associated with university aspirations through increased school satisfaction (Vernon et al., 2019). And as secondary school aspirations may affect pupils’ later enrolment at university (Klasik, 2012), we proposed that the social experiences in secondary school are connected to enrolment at university through adolescents’ aspirations towards university. Considering the inequalities in university access, we also researched the role of ethnicity. Using a nationally representative longitudinal study in England that followed young people from early to late adolescence, we aimed to investigate how adolescents’ secondary school relationships with teachers or peers impacted their university aspirations and enrolment over 5 years.

We had the following expectations: (1) self‐ (and parent‐) reported experiences of peer victimization in early adolescence are associated with weaker university aspirations and subsequently with a lower likelihood of university enrolment, and these longitudinal effects are mediated via secondary school engagement; (2) adolescents’ experiences of teacher support in early adolescence are associated with higher university aspirations and subsequently with a higher likelihood of university enrolment, and these effects are mediated via secondary school engagement. Given the limited findings on the interaction between teacher support and peer victimization, we also explored whether teacher support would lessen the negative effects of peer victimization. Last, considering the inconsistent findings on how university choices vary by ethnicity, we researched whether the impact of teacher support and peer victimization varied across ethnic minority and majority adolescents.

## METHOD

### Participants

The sample was drawn from a longitudinal study of young people in England (Next steps, University College London, 2021). We used the first three annual waves, Times 1–3 (T1–T2–T3) corresponding to Years 9–10–11 of English secondary education (academic age 13–14–15 years). We also included a question about university enrolment from the sixth wave (T4), matching the first year in the UK Higher Education (age 18 years). We excluded those with missing data on ethnicity and those from certain ethnic groups that were too small to analyse.^1^ In the final sample (*N* = 15,110; Male: 51%; Female: 49%), most adolescents self‐identified as White^2^ (68%; mainly White British). We categorized minority groups as Black (12%; mainly African, Caribbean or mixed) and Asian (20%; mainly from South‐Asian backgrounds, Indian, Pakistani or Bangladeshi). The aggregated groups were following the categorization such as the one by the DfE (2021), apart from including mixed background pupils into relevant minority groups. This is because of the evidence suggesting that mixed‐race pupils’ experiences are similar to their minority counterparts (Campion, 2019). Please note that we applied weights, and this affected the size of ethnic groups in our analyses. Supplementary Online Materials show the ethnic composition of each group. Data from T1‐ to T3 were collected via face‐to‐face interviews in the respondent's home, including an interview with the young person and their parents/guardians. Information about peer victimization, teacher support and school engagement was obtained via a self‐completed computer questionnaire; about half of adolescents at T1 (45%) completed this with no one else in the room, in 54% of our final sample there was at least one person present in the room (e.g., parent or sibling). T4 collection involved an interview with the young person only, conducted online, over the telephone or face‐to‐face (for sampling and data collection, see the technical report; DfE, 2011).

### Measures

The following variables have been modelled as latent variables in all the models reported under Results. Latent variables can be understood as continuous, normally distributed variables, where positive values indicate above mean values and negative below mean values.

***Teacher support*** was measured with three items from the ‘attitudes to school/teachers’ section (DfE, 2011): “I like my teachers” and “My teachers praise me when I do my schoolwork well,” both coded at a 5‐point ordinal scale from 1 = *none of my teachers* to 5 = *all of my teachers*, and a binary item (re‐coded from 3‐point item) where 1 = *Most of my teachers try hard to make me work as well as I am able* and 0 = *Most teachers are fairly easily satisfied*/*Don't seem to care whether I work or not*. This item was collapsed as only 3.5% of pupils selected “don't seem to care” and we believe that the latter two options (easily satisfied–does not care) were not worded in a distinguishable way, such as some pupils may perceive that being easily satisfied and showing a lack of care are interchangeable. These items were previously included in a measure of ‘perception of teacher behaviour/professionalism’ (Attwood & Croll, 2011). Similar to established measures of perceived teacher support (e.g., Malecki & Demaray, 2002; Metheny et al., 2008; Torsheim et al., 2000), our measure focused on affective support such as teachers’ praise or positive regard, and teachers’ interest in adolescents’ work. Due to low number of items using different scoring, reliability was assessed in terms of interpretability and interitem correlations, *r*_min_ = .31, *r*_max_ = .42, *p* < .001. The confirmatory factor analysis (CFA) showed that all items loaded strongly (λ_standardized_ > .59) on the underlying factor (teacher support).

#### Peer victimization

Self‐reported peer victimization was measured by five binary (0 = *no*, 1 = *yes*) items transformed into a latent variable, where higher values indicate more peer victimization experiences. The items focused on experiences with hurtful name calling, exclusion, threats, physical violence and having a possession taken by other pupils in school in the last 12 months (e.g., “have you ever been excluded from a group of friends or from joining in activities?” and “have other students ever threatened to hit you, kick you or use any other form of violence against you?”). The CFA showed that all items loaded strongly (λ_standardized_ > .52) on the underlying factor (peer victimization). As methods such as McDonald's omega are not suitable for assessing reliability with binary items, we evaluated how well the factor was measured by considering the item information curves (see Supplementary Online Materials). To establish whether the examined effects of self‐reported peer victimization remained comparable, we replicated our models using parent‐reported peer victimization, obtaining comparable results (see Supplementary Online Materials for details).

***School engagement*** was measured with 11 items from the ‘attitudes to school/teachers’ section of the questionnaire (DfE, 2011), such as “I am happy when I am at school” and “School is a waste of time for me (recoded).” These items were used as a “feelings about school” scale (Bowe, 2020), and a subsection of these items as a measure of emotional school engagement (Gutman & Schoon, 2018). When compared to established measures of school engagement for adolescents (see Fredricks et al., 2011), our measure focused on similar aspects of engagement such as valuing school or enjoying schoolwork. Responses utilized a 4‐point scale from 1 (*strongly disagree*) to 4 (*strongly agree*). The scale was reliable, T1–T2 school engagement McDonald's ω = .81–.84. CFA showed a good fit for both T1 and T2 school engagement, RMSA = .05 and CFI = .95, establishing their validity.

The following outcome variables have been measured as a single item:

***University aspirations*** were based on one question: “How likely do you think it is that you will ever apply to go to university to do a degree?” scored from 1 (*not at all*) to 4 (*very likely*), previously used as a measure of aspirations (Croll & Attwood, 2013) and in combination with other items as a measure of educational expectations (Khattab, 2015; Lazarus & Khattab, 2018). We used this selected item as a measure of university aspirations, distinct from expectations, as a wish to apply to university does not mean that the young person expects to succeed.

***University enrolment*** was based on a binary item asking the young person whether they were currently (at T4) doing a course at university, coded as 1 (*yes*) and 0 (*no*).

#### Control variables

We controlled for young person's gender (0 = *Male*, 1 = *Female*) and household socio‐economic status (SES) defined as a latent factor with two items: parental education and household salary, as SES is an important predictor of university choices (Nieuwenhuis et al., 2019).

### Analyses

#### Model specification

We ran a longitudinal structural equation model (SEM) in Mplus 8 (Muthén & Muthén, 2017) using a robust maximum likelihood (MLR) estimator. The advantage of the SEM model is that it allows for the estimation of latent variables (from observed variables), and it takes into account the measurement error (Kline, 2016). To deal with sample attrition, we used all available data and applied T1 weights (for weights, see the technical report, DfE, 2011). We also took into account clustering and stratification (where each cluster represented one school and strata were based on area deprivation). We, therefore, specified both main‐effect and interaction SEM as complex type analysis with the Monte Carlo integration algorithm. The advantage of using MLR with the Monte Carlo integration is that it can be used to model interactions between latent variables, and we could therefore test whether including interactions in the model improved the fit. We would like to highlight that in large samples such as the one used in this study, even very small effects tend to be detectable (significant), and therefore we did not solely rely on statistical significance when interpreting our results, but we also considered their theoretical and practical justification. We also applied a stricter alpha level, that is, *p* < .01.

#### Procedure

We followed these two steps to test the main effect and the interaction models. First, we analysed the effects of teacher support, peer victimization and ethnicity on university aspirations (via T1–T2 school engagement) and enrolment (via T1–T2 engagement and T3 university aspirations). T1 and T2 school engagement were considered as serial mediators (i.e., T1 engagement predicting T2 engagement). We examined both direct and indirect (mediated) effects. Second, we included the interactions among teacher support, peer victimization and ethnicity, to explore whether (1) teacher support moderated the effect of peer victimization on school outcomes (compensatory expectation); and (2) peer victimization and (3) teacher support effects on school outcomes varied across ethnic groups. In both models, we controlled for the effects of gender and SES.

## RESULTS

Table 1 reports descriptive statistics. Looking at the response distribution for items defining the latent variables, we found that adolescents overall reported positive engagement, high teacher support and high university aspirations. Almost half of our sample (46%) experienced peer victimization at T1.

**TABLE 1:** Descriptive statistics and correlations for all study variables

	*M* (*SD*)/%	1	2	3	4	5	6	7	8	9
*Latent variables*										
1. School engagement T1	.00 (.47)									
2. School engagement T2	.00 (.48)	.78***								
3. Teacher support T1	.00 (.59)	.77***	.59***							
4. Peer victimization T1	.00 (1.51)	−.18***	−.18***	−.17***						
5. SES	.00 (1.31)	.12***	.14***	.06***	−.02					
*Manifested variables*										
6. University aspirations T3	2.78 (1.14)	.36***	.43***	.22***	−.11***	.31***				
7. University enrolment T4	‘yes’ 32%	.25***	.29***	.17***	−.11***	.26***	.48***			
8. Asian ethnicity	‘Asian’ 20%	.12***	.11***	.06***	−.10***	−.17**	.16***	.09***		
9. Black ethnicity	‘Black’ 12%	.05***	.03**	−.02	−.02	−.02	.10***	.01	−.06***	
10. Gender	‘Female’ 49%	.06***	.05***	−.05***	−.04**	.01	.14***	.07***	.01	.02

Note

Correlations, means and *SD* for latent variables are based on estimates from a simpler SEM model (without regression effects) in which we modelled covariances between all variables. The reference categories are ‘not attending university’, male and White ethnicity.

**p < .01; ***p < .001.

### Establishing latent variables

To confirm that all latent variables used in our final model described their underlying factors well, we first ran a CFA using all latent variables, that is, school engagement (T1–T2), teacher support, peer victimization and SES, allowing bivariate correlations between them. The CFA showed that all standardized factor loadings were strong (λ_standardized_ > .40) and statistically significant, *p* < .001. We then expanded this model by including the directional relationships between our predictor and outcome variables (main effect model) and finally by including the interactions. The standardized factor loadings reported in Table 2 were based on the results from the interaction model.

**TABLE 2:** Standardized factor loadings and covariances for the CFA

School engagement	T1		T2	
	λ	*SE*	λ	*SE*
On the whole I like being at school	.67	.01	.67	.01
I am happy when I am at school	.57	.01	.60	.01
School is a waste of time for me (recoded)	.53	.01	.60	.01
School work is worth doing	.46	.01	.41	.01
Most of the time I don't want to go to school (recoded)	.65	.01	.67	.01
I work as hard as I can in school	.52	.01	.53	.01
In a lesson, I often count the minutes till it ends (recoded)	.46	.01	.48	.01
I am bored in lessons (recoded)	.69	.01	.69	.01
The work I do in lessons is a waste of time (recoded)	.58	.01	.60	.01
The work I do in lessons is interesting to me	.63	.01	.61	.01
I get good marks for my work	.43	.01	.45	.01
*Between‐item residual covariances*				
I am happy when I am at school. WITH On the whole I like being in school	.35	.01	.41	.01
I get good marks for my work. WITH I work as hard as I can in school	.21	.01	.23	.01
I am bored in lessons. WITH In a lesson, I often count the minutes till it ends	.27	.01	.34	.01
The work I do in lessons is a waste of time WITH School is a waste of time for me	.20	.01	.20	.02
I like my teachers	.75	.01		
My teachers praise me when I do my schoolwork well	.54	.01		
Most of my teachers try hard to make me work as well as I am able/Are fairly easily satisfied OR Don't seem to care whether I work or not	.58	.01		

Peer victimization (self‐report) T1				
In the last 12 months, have…				
You ever been upset by being called hurtful names by other students, including getting text messages or emails from them?	.68	.01		
You ever been excluded from a group of friends or from joining in activities?	.53	.01		
Other students ever ACTUALLY hit you, kicked you or used any other form of violence against you?	.83	.01		
Other students at your school ever made you give them money or personal possessions?	.63	.02		
Other students ever THREATENED to hit you, kick you or use any other form of violence against you?	.94	.01		

Household socio‐economic status				
Household estimated yearly salary	.42	.02		
Highest qualification – parent	.99	.00		

Between factor covariances		*p*		
T1 teacher support* T1 peer victimization	−.17	<.001		
T1 teacher support* Household SES	.08	<.001		
T1 peer victimization* Household SES	−.03	.008		

Note

All reported standardized loadings, and between‐item residual standardized covariances were significant at *p* < .001. Effects are based on the interaction SEM. Some items in the school engagement scales shared a residual covariance which was not explained by the factor. These covariances were included in the model to improve the fit. We reported StdYX standardization, which standardizes both the predictor and the outcome by the standard deviation.

### Main effects: peer victimization, teacher support and ethnicity

The SEM model examining the main effects of peer victimization, teacher support and ethnicity explained a large proportion of variance in school outcomes (Table 3). Excluding the control variables gender and SES, teacher support and peer victimization together explained around 60% of the variance in T1 engagement (note that victimization alone explained around 3% of the variances in T1 engagement), 61% in T2 engagement, 19% in T3 aspirations and 4% in T4 enrolment. We provided the coefficients for the direct effects in Table 3 and reported the indirect effects below.

**TABLE 3:** Main effects model: School outcomes predicted by self‐reported peer victimization, teacher support and ethnicity

	Dependent variable	T1 School engagement				T2 School engagement				T3 University aspirations				T4 University enrolment			
		*b*	(*SE*)	*p*	*β*	*b*	(*SE*)	*p*	*β*	*b*	(*SE*)	*p*	*β*	logit *b*	(*SE*)	*p*	*OR*
Main effects	Peer victimization	−.01	(.00)	.008	−.03	−.01	(.00)	.001	−.04	−.01	(.01)	.556	−.01	−.08	(.03)	.003	.92
	Teacher support	.59	(.01)	<.001	.76	−.01	(.02)	.635	−.01	−.01	(.03)	.814	.00	.39	(.07)	<.001	1.48
	Asian ethnicity	.18	(.01)	<.001	.38	.06	(.01)	<.001	.12	.77	(.03)	<.001	.66	.39	(.10)	<.001	1.47
	Black ethnicity	.13	(.01)	<.001	.28	−.02	(.02)	.325	−.03	.54	(.03)	<.001	.46	−.23	(.12)	.054	.80
Mediators (serial)	T1 School engagement					.77	(.03)	<.001	.77								
	T2 School engagement									.80	(.04)	<.001	.33				
	University aspirations													1.07	(.04)	<.001	2.90
Controls	Household SES	.03	(.00)	<.001	.11	.02	(.00)	<.001	.07	.28	(.01)	<.001	.37	.31	(.04)	<.001	1.36
	Gender	.07	(.01)	<.001	.15	.01	(.01)	.317	.02	.27	(.03)	<.001	.23	.09	(.07)	.155	1.10
	*R* ^2^	.63				.61				.36				.44			

Note

Table reports the unstandardized regression coefficients with their standard errors, standardized regression coefficients and the odds ratio (OR) for the categorical outcome. The standardized coefficient represents a change in outcome (in SD units) when the predictor variable increases by 1 SD (for continuous)/changes from 0 to 1 (for binary). Peer victimization, teacher support, SES and school engagement were defined as latent variables.

#### Peer victimization

As expected, peer victimization had a small, statistically significant negative effect on school outcomes. At T1, peer victimization was related to lower school engagement. Peer‐victimization (T1) subsequently led to lower school engagement the following year at T2, both directly and indirectly through lower T1 engagement, *b*_total indirect_ = −.01, *SE* = .00, *p* = .007. Peer victimization at T1 was also indirectly associated with lower university aspirations at T3 via decreased school engagement at T1 and T2, *b*_total indirect_ = −.02, *SE* = .00, *p* < .001.

Moreover, peer victimization at T1 also decreased the likelihood of attending university 5 years later at T4, both directly and indirectly via lower school engagement at T1, T2 and, in turn, lower aspirations at T3 (serial mediation), *b*_specific indirect_ = −.01, *SE* = .00, *p* = .010. Engagement was a critical mediator, as the effect of peer victimization on university enrolment was not mediated solely via university aspirations at T3 (i.e., when T1 and T2 engagement were not included as mediators), *p* = .556. The odds ratio shows that peer victimization decreased the likelihood of university enrolment. For every 10 adolescents in the reference group (White adolescents with mean values of university aspirations, SES, teacher support and peer victimization), there would be only 9 adolescents attending university if their peer victimization increased by 1 while all other adolescent characteristics/predictors stayed constant. Our results suggest that due to the indirect effects of peer victimization, the total decrease in likelihood was even more substantial. Overall, peer victimization at T1 led to worse school outcomes in the following years directly and indirectly through decreased school engagement.

#### Teacher support

Teacher support at T1 was associated with higher school engagement at T1, which in turn led to higher school engagement the following year at T2, *b*_total indirect_ = .46, *SE* = .02, *p* < .001. This effect was fully mediated via T1 engagement, as the direct effect of T1 teacher support on T2 engagement was not statistically significant, *p* = .635. Teacher support at T1 was also related to higher university aspirations at T3, and this effect was fully mediated through school engagement at T1 and T2 as serial mediators, *b*_specific indirect_ = .37, *SE* = .02, *p* < .001. School engagement at T1 was the critical mediator as T2 school engagement alone was not a statistically significant mediator, *p* = .635.

Higher teacher support at T1 was also related to a higher likelihood of attending university at T4, both directly and indirectly via higher school engagement at T1, leading to increased school engagement at T2 and in turn higher aspirations at T3, *b*_specific indirect_ = .39, *SE* = .03, *p* < .001. The indirect effect of teacher support on enrolment was significant only when all three serial mediators were included (specific indirect effects of university aspirations *p* = .814 and university aspirations with T2 engagement *p* = .635), meaning that T1 engagement had a critical role in mediating teacher support with university enrolment. To interpret, adolescents who scored 1 on teacher support were 1.5 times more likely to enrol at university at T4. Our results suggest that the total increase in enrolment odds was even higher due to the indirect effect of teacher support. Overall, higher teacher support at T1 was related to more positive school outcomes in the following years, and these effects were mediated via school engagement.

#### Ethnicity

Asian or Black minority adolescents had higher school engagement at T1 (and T2 for the Asian group only) and higher university aspirations at T3 compared to White majority adolescents. Asian minority pupils also had a higher likelihood of university enrolment at T4, both directly and indirectly through increased engagement at T1–T2 and aspirations at T3, *b*_total indirect_ = .99, *SE* = .05, *p* < .001. We did not find a significant direct effect for the Black group, however, Black adolescents overall had a higher likelihood of attending university indirectly, through increased aspirations at T3, as well as increased T1–T2 engagement, *b*_total indirect_ = .65, *SE* = .05, *p* < .001.

### Interactions among teacher support, peer victimization and ethnicity

In the next step, we modelled the effects of interactions on T1 school engagement (as the mediator of T2–T3–T4 outcomes) to explore whether teacher support lessened the negative impact of peer victimization, and whether the effects of teacher support and peer victimization differed across ethnic minority and majority adolescents. Including the interactions improved the model fit as the Akaike and Bayesian information criterion yielded lower values. While we focus on T1 engagement in this study, the results of our main effects model showed that school engagement did not fully mediate the path to university choices. We, therefore, also modelled and reported the interaction effects on T2–T3–T4 school outcomes in the Supplementary Online Materials. We only kept the significant interactions in the model.

**FIGURE 2:**
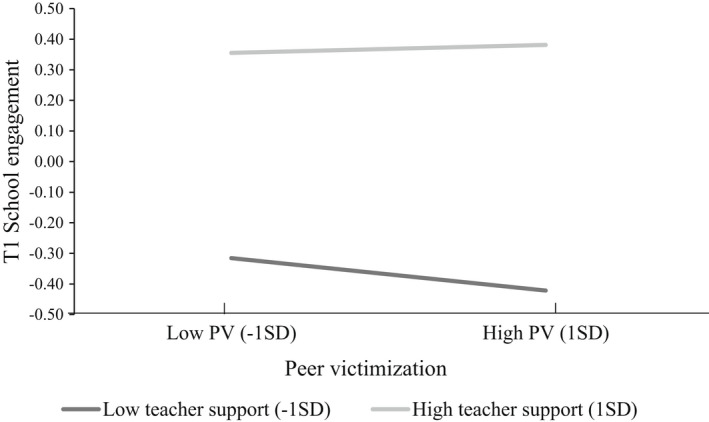
School engagement of adolescents with high versus low teacher support at T1, conditional on peer victimization experiences. *Note*. Simple slopes significant at *p* < .001

Results shown in Table 4 suggested that teacher support might indeed compensate for the negative effect of peer victimization. As shown in Figure 2, the positive association between teacher support and school engagement was slightly stronger for those subjected to more peer victimization than for those subjected to less peer victimization.

**TABLE 4:** Interaction model: School outcomes predicted by interactions among peer victimization, teacher support and ethnicity

	Dependent variable	T1 School engagement			T2 School engagement			T3 University aspirations			T4 University enrolment			
		*b*	(*SE)*	*p*	*b*	(*SE*)	*p*	*b*	(*SE*)	*p*	*B*	(*SE*)	*p*	*OR*
	Peer victimization	−.01	.00	.002	−.01	.00	.001	−.01	.01	.470	−.08	.03	.001	.92
	Teacher support	.60	.01	<.001	−.01	.02	.536	−.02	.03	.479	.37	.07	<.001	1.45
	Asian ethnicity	.19	.01	<.001	.06	.01	<.001	.75	.03	<.001	.33	.09	<.001	1.39
	Black ethnicity	.13	.01	<.001	−.02	.02	.285	.53	.03	<.001	−.27	.12	.021	.77
	Peer victimization*Teacher support	.03	.01	<.001										
	Teacher support*Asian ethnicity	−.16	.02	<.001										
	Teacher support*Black ethnicity	−.06	.02	.003										
Mediators (serial)	T1 School engagement				.78	.03	<.001							
	T2 School engagement							.86	.04	<.001				
	University aspirations										1.11	.04	<.001	3.05
Controls	Household SES	.03	.00	<.001	.02	.00	<.001	.26	.02	<.001	.27	.03	<.001	1.31
	Gender	.07	.01	<.001	.01	.01	.343	.27	.03	<.001	.07	.07	.254	1.08
	*R* ^2^	.64			.61			.33			.45			

Note

Table reports the unstandardized regression coefficients with their standard errors, standardized regression coefficients and the odds ratio (OR) for the categorical outcome. The standardized coefficient represents a change in outcome (in SD units) when the predictor variable increases by 1 SD (for continuous)/changes from 0 to 1 (for binary). Peer victimization, teacher support, SES and school engagement were defined as latent variables.

**FIGURE 3:**
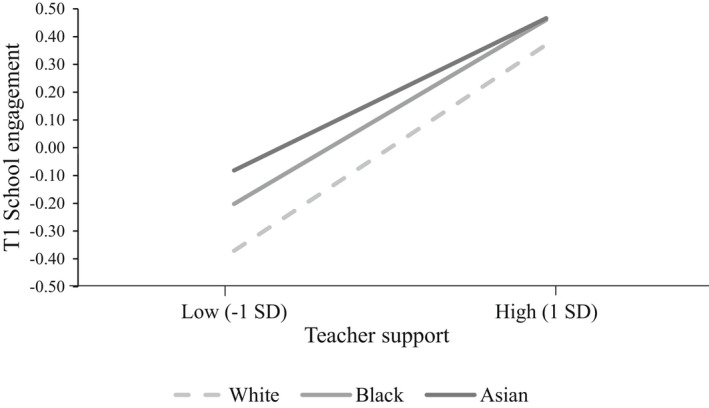
School engagement of adolescents with high versus low teacher support at T1, conditional on ethnicity. *Note*. Simple slopes significant at *p* < .001

The two significant interactions between ethnicity and teacher support were summarized in Figure 3. It shows that the positive association between teacher support and school engagement was stronger for White adolescents than it was for Asian and Black minority adolescents. In other words, despite having higher T1 school engagement overall, Asian and Black minority adolescents benefited less from the positive effects of high teacher support on T1 school engagement compared to White majority adolescents.

We provide the diagram of all significant paths in the interactions model with standardized coefficients in Supplementary Online Materials.

## DISCUSSION

Using a longitudinal study that extended over 5 years, we investigated how ethnic minority and majority adolescents’ individual relationships with their peers and teachers in secondary school impact their path to university. We are the first study, to our knowledge, to show the longitudinal impact of peer victimization and teacher support in early adolescence on later university enrolment using a nationally representative sample from Europe. Thus, our findings shed light on the role of the social ecology of ethnic minority and majority adolescents’ school environments in their (unequal) access to higher education (Richardson, 2015; UCAS, 2020). In our proposed model, we hypothesized that social relationships in secondary school would be connected to university aspirations and enrolment through secondary school engagement, and our findings concerning the role of the social environment at the micro‐system (peer victimization and teacher support) and macro‐system (ethnic background) support this model.

### Peer victimization

Investigating the long‐term effects of peer victimization in secondary school, we found that peer victimization had a small, statistically significant negative relationship to school outcomes in line with previous studies (Nakamoto & Schwartz, 2010). Going beyond previous research, we also showed that these associations were long lasting, as those who experienced peer victimization at age 13 reported lower university aspirations 2 years later and had a lower probability of attending university at 18 years. These associations were mediated via school engagement at T1 and T2 (at ages 13 and 14), highlighting the key role of school engagement developed during the early years of secondary education as the connection between negative peer relationships and school outcomes (Buhs, 2005; Nakamoto & Schwartz, 2011; Roopa et al., 2010; Zimmer‐Gembeck et al., 2006). Furthermore, the negative effects of peer victimization were present for ethnic minority and majority adolescents likewise, as we did not find any strong evidence that ethnic group membership moderated the effects of peer victimization. However, it is important to acknowledge that peer victimization was associated with adolescents’ outcomes only weakly, suggesting that other social experiences may be more detrimental for adolescents’ academic outcomes.

### Teacher support

Focusing on adolescents’ positive relationships with their teachers in secondary school, we found that in accordance with the previous studies (Ahmed et al., 2010; Garcia‐Reid et al., 2015; Roorda et al., 2017), perceived teacher support was beneficial for school outcomes. Adding to the extensive evidence on the importance of student–teacher relationships for other school outcomes (McGrath & Van Bergen, 2015; Roorda et al., 2017; Sabol & Pianta, 2012), our findings provide a unique insight into the role of teacher support in university choices. Those who perceived their secondary school teachers as supportive reported higher university aspirations 2 years later and were more likely to attend university 5 years later. These associations were mediated via school engagement during the early years of secondary education. Thus, teacher support was associated with higher school engagement, and in turn better school outcomes in the forthcoming years. These associations not only persisted over several years but also explained a large variance, particularly in school engagement. Roorda et al. (2011) found that ethnic minority adolescents may benefit more strongly from the positive effects of teacher support. However, our findings suggested the opposite: Black and particularly Asian minority adolescents seemed to benefit less from teacher support. Taken with the findings of under‐attainment among some minority students in the UK higher education (Richardson, 2015), our results suggest that minority adolescents might be facing additional barriers on their path to university, such as not benefiting as much from supportive resources. However, we note that these differences, while statistically significant, were small.

### Ethnicity

Considering environmental influences beyond immediate social relationships (Bronfenbrenner, 1977), we also investigated the role of ethnicity. Controlling for SES, ethnic minority (Black and south Asian) adolescents reported higher engagement in secondary school and higher aspirations to apply to university than their White peers. Through higher engagement, minority ethnicity adolescents had (indirectly) a higher probability of university enrolment, providing some clarification on the low university enrolment rates among Whites compared to minority groups (UCAS, 2020). However, Asian minority pupils had simultaneously a higher *direct* likelihood of attending university compared to majority (White) peers, indicating that there are other reasons for some ethnic groups to have a higher likelihood of entering university, which could not be explained just by their higher aspirations.

### Socio‐ecological interactions

Finally, in line with the ecological systems approach and the expectation that protective relationships may compensate for the risk factors in adolescent development (Bronfenbrenner, 1977; McGrath & Van Bergen, 2015; Sabol & Pianta, 2012), our results suggest that teacher support could mitigate the negative associations of peer victimization to school outcomes. Our results show that adolescents who are subjected to more peer victimization experiences and who perceive low support from their teachers in secondary school are particularly vulnerable to lower school engagement; however, this trend can be reversed when teacher support is high.

#### Limitations

Future studies may be able to strengthen the conclusions of this study by overcoming some of its limitations. As this was a secondary analysis, our data were not collected with a focus on school relationships and did not use standardized instruments. For example, the peer victimization scale did not include an instruction clarifying the peer victimization context and frequency was only asked as a follow‐up question. Although our measures were similar to established measures in content and validated through CFAs, future studies should examine if these findings replicate with standardized measures. Next, although longitudinal, this study used a correlational survey that does not allow for testing causality. Since most measures were not repeated over the years, it was not possible to test the bidirectionality of these associations either. Creating the aggregated ethnic groups also had its limitations, as we found disparities in university enrolment among ethnic subgroups used for all aggregate groups (White, Black and Asian). Finally, this study only examined either the positive or the negative aspect of peer and student–teacher relationships. Future studies should test both positive and negative aspects simultaneously, such as by including peer support measures. Future studies should also consider other micro‐systems, as parents may also impact adolescents’ enrolment through engagement and aspirations (Hill & Wang, 2015). While these limitations should be considered when interpreting the results, we believe that the benefits of using a large, nationally representative and longitudinal dataset outweigh its limitations.

## CONCLUSION

In summary, our results indicate that both negative and positive social experiences of adolescents during their early years of secondary school have a long‐lasting effect on their path to university through engagement in secondary school. Our findings suggest inequalities in university access do not necessarily stem from the lack of positive resources perceived by minority pupils, as their relationships with teachers and engagement in school were generally positive, but rather from how they benefit from these resources. In conclusion, it is vital to perceive the school environment not only as a learning institution but also as a system influenced by various social and educational experiences that shape adolescents’ future outcomes and choices.

## CONFLICT OF INTEREST

All authors declare no conflict of interest.

## AUTHOR CONTRIBUTION

**Eva Grew:** Conceptualization; Formal analysis; Methodology; Project administration; Visualization; Writing – original draft; Writing – review & editing. **Gülseli Baysu:** Conceptualization; Formal analysis; Methodology; Project administration; Resources; Supervision; Writing – review & editing. **Rhiannon N. Turner:** Conceptualization; Supervision; Writing – review & editing.

## Data Availability

The data that support the findings of this study are openly available in UK Data Service at http://doi.org/10.5255/UKDA‐SN‐5545‐7 [doi], 5545 [reference number].

## Footnotes

^1^
Participants who self‐identified as “Chinese” (*n* = 44), “Any other mixed background” (*n* = 128) or “Any other” (*n* = 130) were excluded. Chinese minority group was excluded from the aggregate Asian group since its distinctive school achievement in the United Kingdom: 80% of Chinese pupils obtained top grade in English and maths, compared to 58% in the aggregate Asian group (DfE, 2021).

^2^
Ethnic background in this study refers to both ethnic and racial backgrounds/groups in line with the UK census categories.
