# Supplementary material for: Experiences of peer victimization and teacher support in secondary school predict university enrolment 5 years later: Role of school engagement
Source: Br J Educ Psychol. 2022 Mar 25;92(4):1295–314. doi: 10.1111/bjep.12500 (PMC9790406; doi:10.1111/bjep.12500)
Supplement: Supplementary file 1 [file BJEP-92-1295-s001.docx]

# Supplementary Online Materials

Additional Descriptive Information

In Table 1s, we provide the means, SD, and correlations between all variables measured at manifested level, i.e., T1-T2 School engagement, teacher support, peer victimization, and SES were calculated as mean scores, rather than modelling them as latent variables.

Table 1s Descriptive statistics for all study variables defined at manifested level

|  | *M* (*SD*)/% | 1 | 2 | 3 | 4 | 5 | 6 | 7 | 8 | 9 |
| --- | --- | --- | --- | --- | --- | --- | --- | --- | --- | --- |
| *Manifested variables* |  |  |  |  |  |  |  |  |  |  |
| 1. School engagement T1 | 3.04 (0.46) |  |  |  |  |  |  |  |  |  |
| 2. School engagement T2 | 2.93 (0.47) | .64^***^ |  |  |  |  |  |  |  |  |
| 3. Teacher support T1 | 0.66 (0.23) | .51^***^ | .39^***^ |  |  |  |  |  |  |  |
| 4. Peer victimization T1 | 0.18 (0.25) | -.13^***^ | -.13^***^ | -.11^***^ |  |  |  |  |  |  |
| 5. SES | 4.11 (2.08) | .10^***^ | .12^***^ | .06^***^ | -.04^***^ |  |  |  |  |  |
| 6. University aspirations T3 | 2.78 (1.14) | .33^***^ | .38^***^ | .16^***^ | -.08^***^ | .29^***^ |  |  |  |  |
| 7. University enrolment T4 | ‘yes’ 32% | .21^***^ | .25^***^ | .12^***^ | -.08^***^ | .24^***^ | .46^***^ |  |  |  |
| 8. Asian ethnicity | ‘Asian’ 20% | .10^***^ | .10^***^ | .04^***^ | -.07^***^ | -.14^***^ | .16^***^ | .08^***^ |  |  |
| 9. Black ethnicity | ‘Black’ 12% | .04^***^ | .02^*^ | -.00 | -.02 | -.03^**^ | .09^***^ | .00 | -.06^***^ |  |
| 10. Gender | ‘Female’ 49% | .05^***^ | .04^***^ | -.03^**^ | -.00 | .01 | .14^***^ | .06^***^ | .01 | 0.02 |

*Note*. ** *p* < .01, *** *p* < .001. The reference categories are ‘*not attending university’*, Male, White ethnicity.

Additional Information on Participants’ Characteristics

We provide detailed information on the ethnic composition of groups in our study (see Table 2s) and the participants’ age. The age variable was not included in the original dataset; however, we were able to work out the age based on information about adolescents’ date of birth and date when the T1 interview was conducted, see Figure 1s. Adolescents in our study were between 10-16 years old, however, only a very small proportion of pupils were less than 13 years old (8 pupils) or more than 14 years old (96). Most students were 13-14 years at the time of the interview (spring), meaning that the vast majority of adolescents was 13 years old at the start of the academic year.

Table 2s A detailed ethnic composition of ethnic groups for the non-weighted sample and after weights were applied

| **Ethnic group:** | ***n*** | ***n*_weighted_** |
| --- | --- | --- |
| **White** |  |  |
| White-British | 10103 | 13003 |
| White-Irish | 29 | 34 |
| Any other White background | 203 | 254 |
| **Black** |  |  |
| African | 613 | 256 |
| Caribbean | 576 | 218 |
| White and Black Caribbean | 394 | 181 |
| White and Black African | 91 | 48 |
| Any other Black background | 91 | 43 |
| **Asian** |  |  |
| Indian | 1013 | 384 |
| Pakistani | 940 | 349 |
| Bangladeshi | 722 | 139 |
| White and Asian | 182 | 121 |
| Any other Asian background | 153 | 128 |

Note. Based on adolescents’ self-designation.

Figure 1s *Age distribution at the time of T1 data collection*

### Additional Information on the Interaction Model

The interaction model presented in our paper used only interactions modelled on T1 engagement, as our expectation was that engagement is a key mediator for the following school outcomes. See Figure 2s for the visual representation of our findings from this model, reporting standardized regression coefficients for all the significant effects (paths) from our analysis (for the unstandardized coefficients and odds ratios, see Table 4 in the main paper).

However, as we also found several direct effects of T1 predictors on T2-T3-T4 academic outcomes, we acknowledge that T1 engagement does not fully mediate these effects, and we have therefore modelled the interactions between teacher support, peer-victimization, and ethnicity on all academic outcomes (T1-T4) in a stepwise fashion, only keeping significant interactions in the model (e.g., all interactions significant when modelled at T1 outcome were kept in the model, and we then modelled interactions on T2). Please note that since this model was computationally demanding and extremely slow due to the number of latent interactions, we had to reduce the number of integration points to 500 (even then the model presented in Table 3s took 16 hours to estimate). However, more integration points would improve the accuracy of the estimates, and we, therefore, recommend some caution for the interpretation of our findings.

This extended interaction model presented in Table 3s supports the findings from our main paper that ethnic minority pupils may benefit less from the positive effect of teacher support on school outcomes, compared to White pupils (see the significant interactions between teacher support and ethnic minorities on T1 engagement, T3 aspirations, and T4 enrolment). In terms of peer victimization, we found it may have a stronger negative effect on majority (White) pupils’ secondary school outcomes, as we found a small positive interaction between ethnic minority groups and peer victimization on T2 school engagement. This model has mixed findings about the idea that teacher support may compensate for the negative effect of peer victimization on academic outcomes. While we found a statistically significant positive interaction between support and victimization on T1 engagement (supporting this idea) we also found a small, statistically significant negative interaction on T3 aspirations, meaning that adolescents who experienced high teacher support and low peer victimization had slightly higher aspirations than those experiencing high teacher support and high peer victimization. In summary, our findings support that positive school relationships may moderate the effect of negative school relationships and that ethnicity may interact with the effects of these relationships. However, we caution against the interpretation of these interactions as the differences were small; and they were direct effects (in the presence of mediators) rather than total effects (without mediators). Interested readers can obtain further information about these interactions from the corresponding author.

Figure 2s


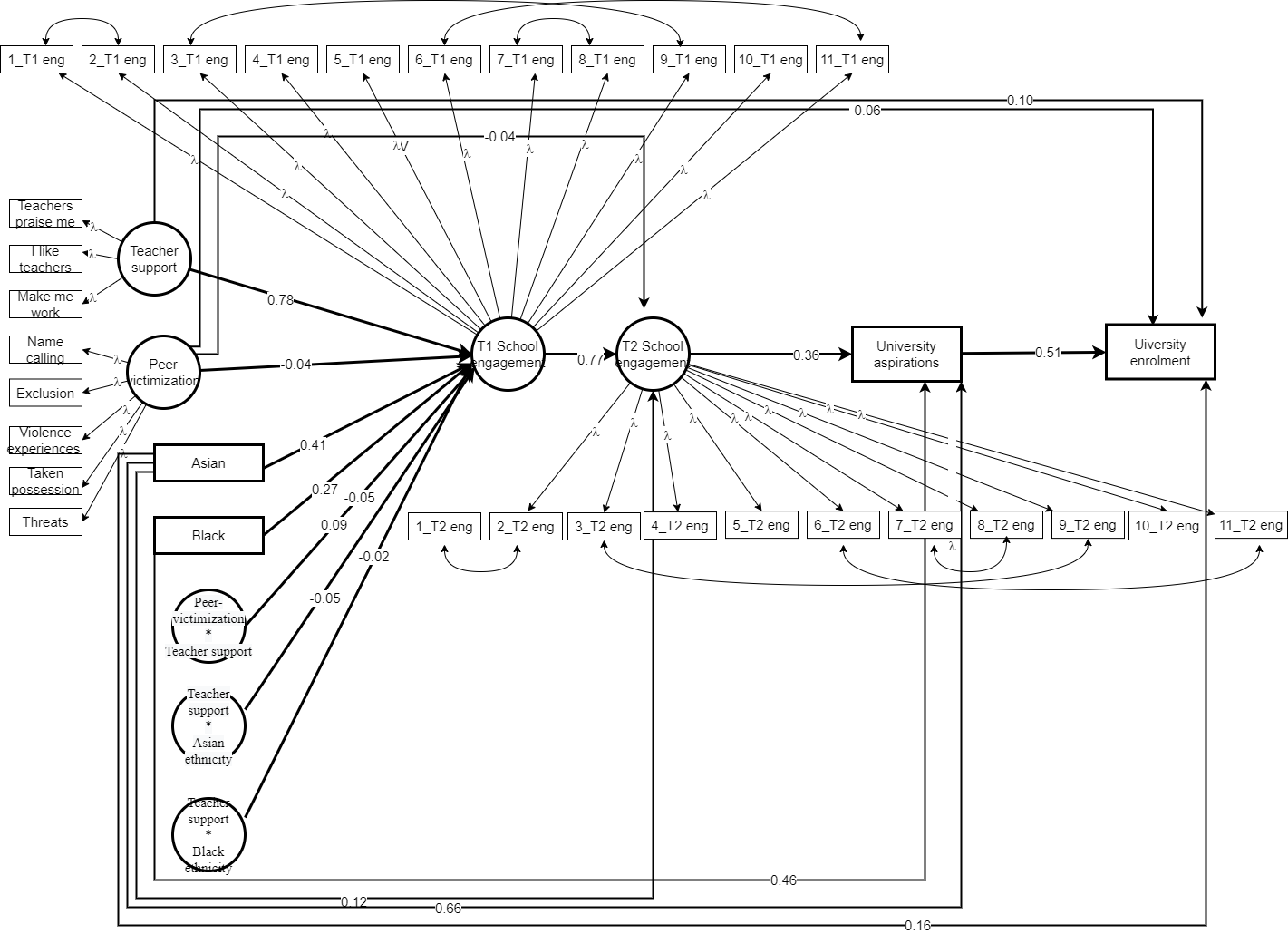
Standardized path coefficients for the interaction model presented in the main paper (Table 4)

Note. Only significant effects were shown. Effects of controls SES and gender omitted in the diagram to improve the readability of the figure. All factor loadings (λ) and covariances can be found in the main paper, Table 2.

Table 3s

Interaction model (interactions on T1-T4): School outcomes predicted by interactions between peer-victimization, teacher support, and ethnicity

|  | *Dependent variable:* | T1 School engagement | | |  | T2 School engagement | | |  | T3 University aspirations | | |  | T4 University enrolment | | |
| --- | --- | --- | --- | --- | --- | --- | --- | --- | --- | --- | --- | --- | --- | --- | --- | --- |
|  |  | *b* | (*SE*) | *p* |  | *b* | (*SE*) | *p* |  | *b* | (*SE*) | *p* |  | B | (SE) | *p* |
|  | Peer-victimization | -0.01 | (0.00) | .009 |  | -0.02 | (0.00) | .001 |  | 0.00 | (0.01) | .891 |  | -0.09 | (0.03) | .014 |
|  | Teacher support | 0.63 | (0.01) | < .001 |  | 0.00 | (0.02) | .979 |  | 0.02 | (0.04) | .534 |  | 0.50 | (0.08) | < .001 |
|  | Asian ethnicity | 0.19 | (0.01) | < .001 |  | 0.06 | (0.01) | < .001 |  | 0.79 | (0.03) | < .001 |  | 0.47 | (0.10) | < .001 |
|  | Black ethnicity | 0.13 | (0.01) | < .001 |  | -0.01 | (0.02) | .577 |  | 0.54 | (0.03) | < .001 |  | -0.17 | (0.12) | .147 |
|  | Peer-victimization*Teacher support | 0.04 | (0.01) | < .001 |  |  |  |  |  | -0.07 | (0.02) | < .001 |  | 0.01 | (0.06) | .933 |
|  | Teacher support*Asian ethnicity | -0.16 | (0.02) | < .001 |  |  |  |  |  | -0.18 | (0.05) | < .001 |  | -0.46 | (0.18) | .013 |
|  | Teacher support*Black ethnicity | -0.06 | (0.02) | .011 |  |  |  |  |  |  |  |  |  | -0.77 | (0.26) | .003 |
|  | Peer-victimization*Asian ethnicity |  |  |  |  | 0.02 | (0.01) | .017 |  |  |  |  |  | -0.05 | (0.09) | .601 |
|  | Peer-victimization*Black ethnicity |  |  |  |  | 0.05 | (0.01) | < .001 |  |  |  |  |  | -0.04 | (0.12) | .767 |
| *Mediators (serial):* | T1 School engagement |  |  |  |  | 0.76 | (0.03) | < .001 |  |  |  |  |  | |  |  |
|  | T2 School engagement |  |  |  |  |  |  |  |  | 0.76 | (0.05) | < .001 |  |  |  |  |
|  | University aspirations |  |  |  |  |  |  |  |  |  |  |  |  | 1.02 | (0.05) | < .001 |
| *Controls* | Household SES | 0.04 | (0.01) | < .001 |  | 0.03 | (0.01) | < .001 |  | 0.35 | (0.02) | < .001 |  | 0.42 | (0.05) | < .001 |
|  | Gender | 0.07 | (0.01) | < .001 |  | 0.01 | (0.01) | .259 |  | 0.28 | (0.03) | < .001 |  | 0.11 | (0.07) | .100 |
|  | *R*^2^ | 0.64 | | |  | 0.61 | | |  | 0.39 | | |  | 0.45 | | |

### Comparisons between CFAs of Parent-Reported and Self-Reported Peer Victimization

In this section, we provide details of the replication of our results based on parent-reported peer victimization. Specifically, we provide details on how we measured parent-reported peer victimization, with a detailed comparison of structures of the parent- and self-reported peer victimization measures as latent factors, as well as information on item characteristic curves. We also provide a detailed comparison of the main effects in the self-reported and parent-reported peer-victimization models.

*Parent-reported peer victimization* is based on parental reports of whether their child experienced peer victimization at school in the last 12 months. Parents were asked to choose each sort of bullying that they knew has happened, the choices comprising of 7 items: “Called names by other pupils at his/her school”, “Sent offensive or hurtful text messages or emails”, “Shut out from groups of other pupils or from joining in things”, “Made to give other pupils his or her money or belongings”, “Threatened by other pupils with being hit or kicked or with other violence”, “Actually being hit or kicked”, “Any other sort of bullying” (1 = *Yes*, 0 = *No*). We defined the parent-reported items at the latent level.

#### Comparison of factor structure in the parent- and self-reported peer victimization

Similar to the CFA (MLR estimator) of self-reported peer victimization (as a single latent factor), CFA with parent reported peer victimization items showed that all the items had a strong relationship to the underlying factor, all standardized factor loadings *λ* > .60 and significant at *p* <. 001. The item response theory analysis (IRT) was used to analyse each item in more detail.

#### Comparison of Item Characteristic Curves in the Parent- and Self-Reported Peer Victimization

In IRT, two characteristics should be considered. First is the steepness of the curve, the steeper the curve the more strongly is the item related to the underlying latent factor, second is the item threshold or difficulty, which is the point where respondent with a given characteristic described as the latent variable (in this case peer victimization) has a 0.5 probability of answering “yes” to a given item (Toland, 2014). To put simply, if we imagine a line at 0.5 probability running through the y-axis in Figures SOM 1-2, the order in which it would “cut” the item characteristic curves indicates the order of how likely the parent/adolescent is to answer “*yes*” if the adolescent is peer-victimized.

The IRT analysis (see Figure 3s) showed that the form of the bullying that the peer-victimized adolescent would be most likely to encounter *according to their parents* was name-calling, followed by threats, actual experience of violence, exclusion, other sorts of bullying and being sent offensive messages. The least likely form of bullying endorsed by parents was being made to hand over money. This is comparable to the results of the IRT for self-reported peer victimization, see Figure 4s.

Overall, the results of the CFA and the IRT for the parent-reported peer victimization were comparable to the self-reported peer victimization reported in the results section.

Figure 3s Item characteristic curves for parent-reported peer victimization items


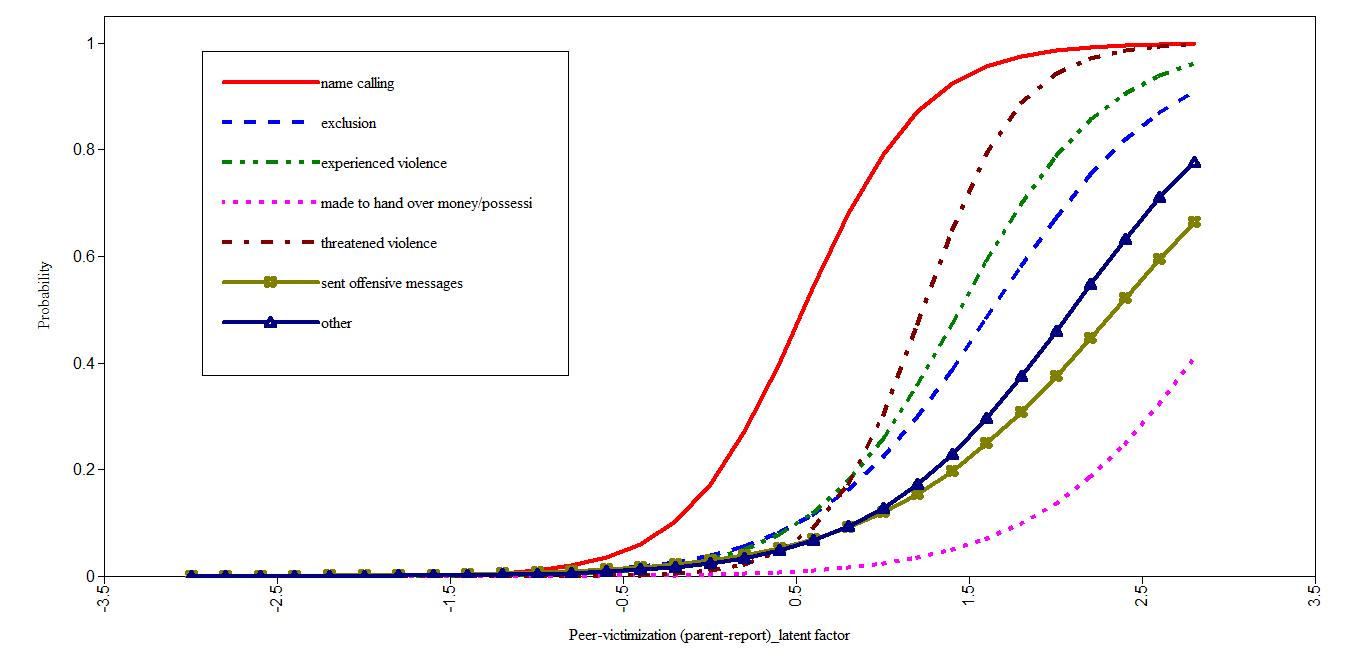


Figure 4s Item characteristic curves for self-reported peer victimization items


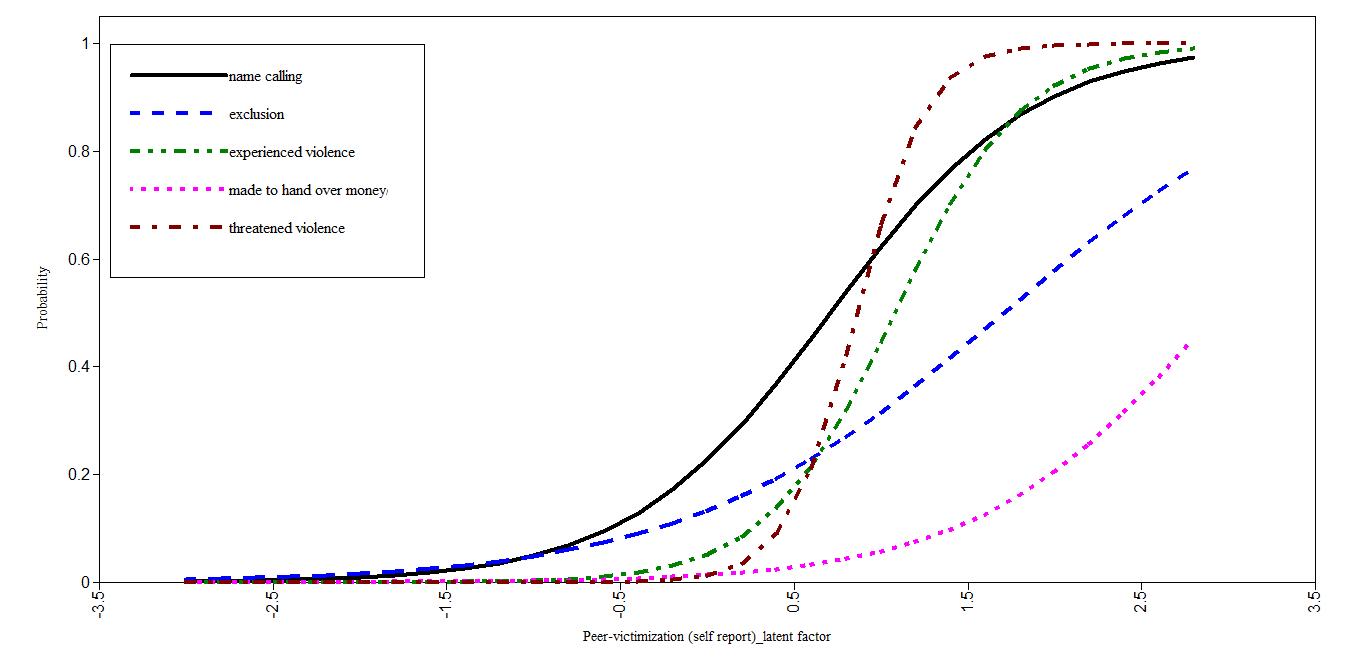


The Item information curves shown in Figure 5s and 6s show that the items do not estimate the peer victimization at the same precision at different levels of peer victimization. In particular, in both parent and self-reported scales, experiencing threats (“threatened violence”) does not estimate peer victimization well at low or very high levels of peer victimization. We should thus use some caution for combining different peer-victimization experiences into one scale, as some items do not estimate peer victimization very well at low levels of peer victimization, and this applies to both parental and self-reported victimization.

Figure 5s Information curves for parent-reported peer victimization items


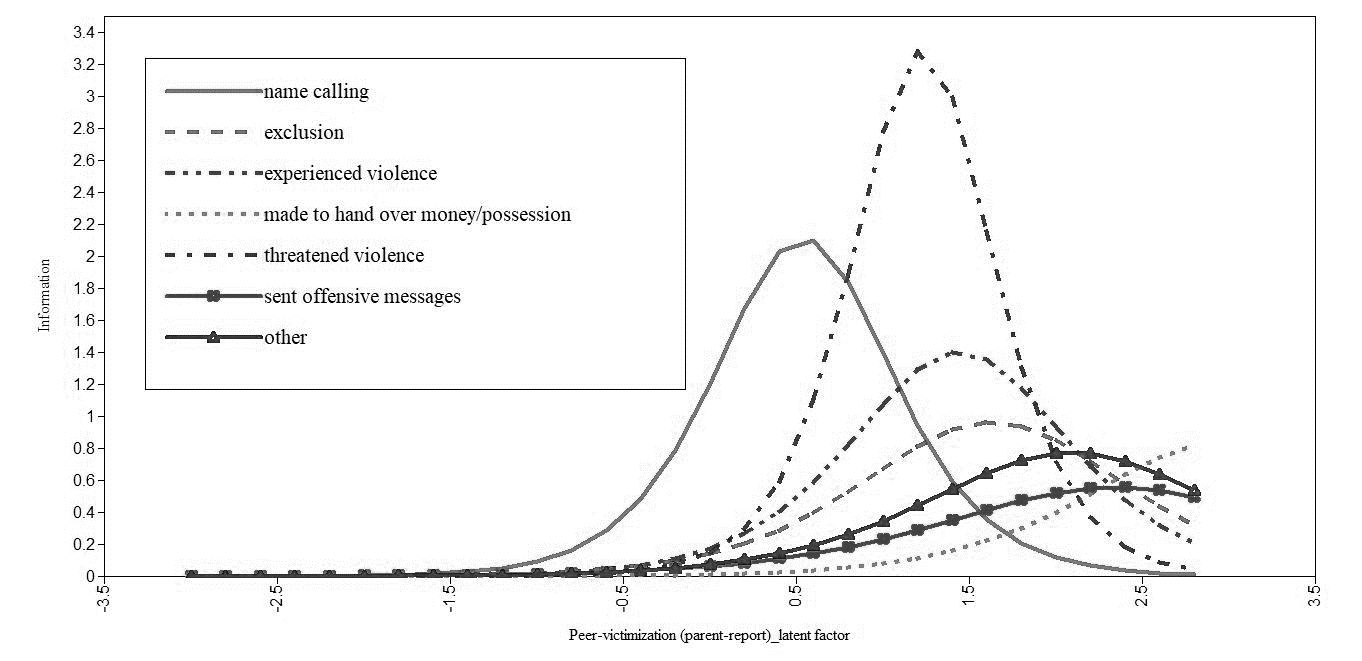


Figure 6s Information curves for self-reported peer victimization items


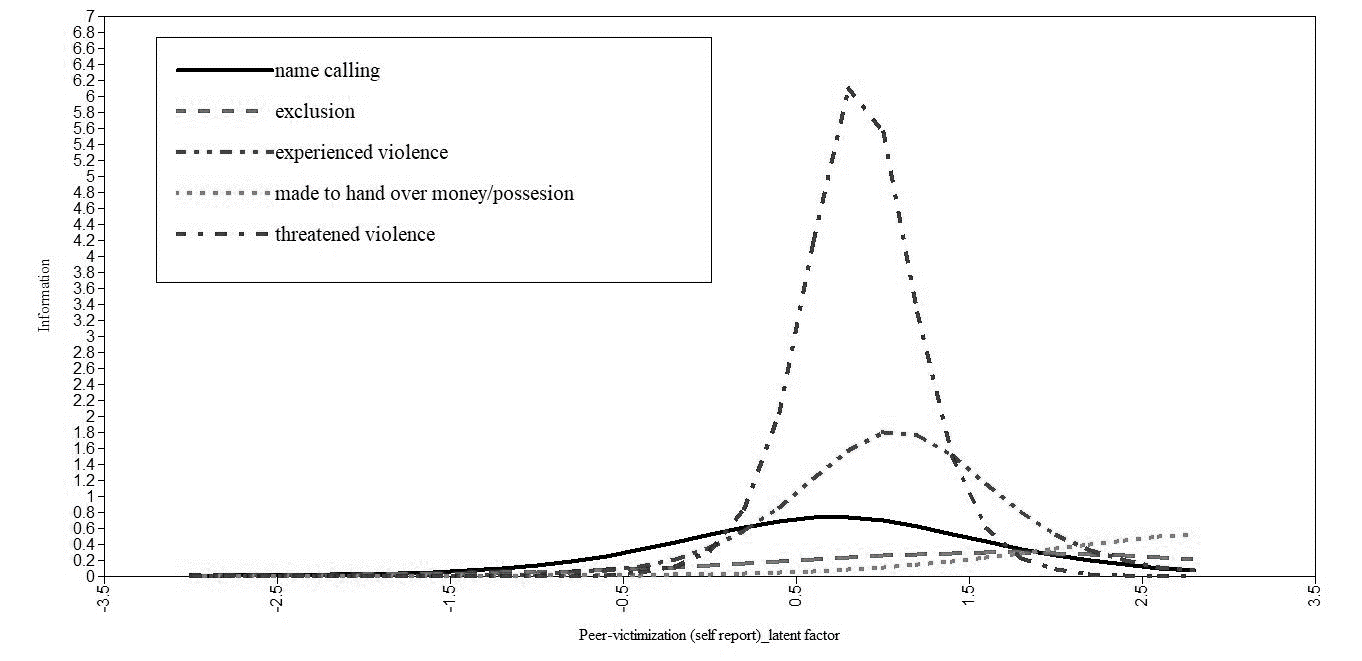


#### Comparisons between Self-Reported and Parent-Reported Peer-Victimization Main Effects Model

Table 4s presents the comparison between the main effect of the self-reported victimization model (also reported under results in the main paper), and the main effects of the parent reported victimization model. In both self- and parent-reported models, peer victimization was measured at latent level, using MLR estimator with Monte Carlo integration algorithm. The effects of peer victimization remained comparable to findings reported in our study (self-report model) even when the information about peer victimization was based on parental report. Thus, peer victimization appears to be related to worse school outcomes regardless of the informant.

One small difference was identified among the models. Peer victimization had a statistically significant direct effect on university aspirations at T3 in the parent-reported model (*p* < .001), but not in the self-reported peer-victimization model. In self-reported model, peer victimization was only related to aspirations indirectly via school engagement. Basing the information about peer victimization on parental report did not change the effects of other predictor and control variables. Overall, the results suggest that the main findings presented in this study are supported in both the self- and parent-reported model of peer victimization.

Table 4s

Comparison between main effects in the self-reported versus parent-reported victimization regression models

|  | Self-reported peer victimization | | | | | | | | | | | | | | | | | | | |
| --- | --- | --- | --- | --- | --- | --- | --- | --- | --- | --- | --- | --- | --- | --- | --- | --- | --- | --- | --- | --- |
|  | *Dependent variable:* | T1 School engagement | | | |  | T2 School engagement | | | |  | T3 University aspirations | | | |  | T4 University enrolment | | | |
|  |  | *b* | (*SE*) | *p* | *β* |  | *b* | (*SE*) | *p* | *β* |  | *b* | (*SE*) | *p* | *β* |  | logit *b* | (*SE*) | *p* | *OR* |
| *Main effects:* | Peer-victimization | -0.01 | (0.00) | .008 | -0.03 |  | -0.01 | (0.00) | .001 | -0.04 |  | -0.01 | (0.01) | .556 | -0.01 |  | -0.08 | (0.03) | .003 | 0.92 |
|  | Teacher support | 0.59 | (0.01) | < .001 | 0.76 |  | -0.01 | (0.02) | .635 | -0.01 |  | -0.01 | (0.03) | .814 | 0.00 |  | 0.39 | (0.07) | < .001 | 1.48 |
|  | Asian ethnicity | 0.18 | (0.01) | < .001 | 0.38 |  | 0.06 | (0.01) | < .001 | 0.12 |  | 0.77 | (0.03) | < .001 | 0.66 |  | 0.39 | (0.10) | < .001 | 1.47 |
|  | Black ethnicity | 0.13 | (0.01) | < .001 | 0.28 |  | -0.02 | (0.02) | .325 | -0.03 |  | 0.54 | (0.03) | < .001 | 0.46 |  | -0.23 | (0.12) | .054 | 0.80 |
| *Mediators (serial):* | T1 School engagement |  |  |  |  |  | 0.77 | (0.03) | < .001 | 0.77 |  |  |  |  |  |  |  |  |  |  |
|  | T2 School engagement |  |  |  |  |  |  |  |  |  |  | 0.80 | (0.04) | < .001 | 0.33 |  |  |  |  |  |
|  | University aspirations |  |  |  |  |  |  |  |  |  |  |  |  |  |  |  | 1.07 | (0.04) | < .001 | 2.90 |
| *Controls* | Household SES | 0.03 | (0.00) | < .001 | 0.11 |  | 0.02 | (0.00) | < .001 | 0.07 |  | 0.28 | (0.01) | < .001 | 0.37 |  | 0.31 | (0.04) | < .001 | 1.36 |
|  | Gender | 0.07 | (0.01) | < .001 | 0.15 |  | 0.01 | (0.01) | .317 | 0.02 |  | 0.27 | (0.03) | < .001 | 0.23 |  | 0.09 | (0.07) | .155 | 1.10 |
|  | *R*^2^ | 0.63 | | | |  | 0.61 | | | |  | 0.36 | | | |  | 0.44 | | | |
| Parent-reported peer victimization | | | | | | | | | | | | | | | | | | | | |
|  | *Dependent variable:* | T1 School engagement | | | |  | T2 School engagement | | | |  | T3 University aspirations | | | |  | T4 University enrolment | | | |
|  |  | *b* | (*SE*) | *p* | *β* |  | *b* | (*SE*) | *p* | *β* |  | *b* | (*SE*) | *p* | *β* |  | logit *b* | (*SE*) | *p* | *OR* |
| *Main effects:* | Peer-victimization | -0.01 | (0.00) | < .001 | -0.04 |  | -0.01 | (0.00) | .001 | -0.04 |  | **-0.03** | **(0.01)** | **< .001** | **-0.08** |  | -0.09 | (0.02) | < .001 | 0.91 |
|  | Teacher support | 0.58 | (0.01) | < .001 | 0.76 |  | -0.01 | (0.02) | .578 | -0.01 |  | -0.01 | (0.03) | .334 | -0.02 |  | 0.38 | (0.07) | < .001 | 1.47 |
|  | Asian ethnicity | 0.18 | (0.01) | < .001 | 0.38 |  | 0.06 | (0.01) | < .001 | 0.12 |  | 0.74 | (0.03) | < .001 | 0.63 |  | 0.32 | (0.09) | < .001 | 1.37 |
|  | Black ethnicity | 0.13 | (0.01) | < .001 | 0.27 |  | -0.02 | (0.02) | .232 | -0.04 |  | 0.52 | (0.03) | < .001 | 0.45 |  | -0.29 | (0.12) | .012 | 0.75 |
| *Mediators (serial):* | T1 School engagement |  |  |  |  |  | 0.78 | (0.03) | < .001 | 0.77 |  |  |  |  |  |  |  |  |  |  |
|  | T2 School engagement |  |  |  |  |  |  |  |  |  |  | 0.85 | (0.04) | < .001 | 0.35 |  |  |  |  |  |
|  | University aspirations |  |  |  |  |  |  |  |  |  |  |  |  |  |  |  | 1.10 | (0.04) | < .001 | 3.01 |
| *Controls* | Household SES | 0.04 | (0.01) | < .001 | 0.09 |  | 0.02 | (0.00) | < .001 | 0.05 |  | 0.33 | (0.02) | < .001 | 0.31 |  | 0.35 | (0.04) | < .001 | 1.42 |
|  | Gender | 0.08 | (0.01) | < .001 | 0.16 |  | 0.01 | (0.01) | .219 | 0.02 |  | 0.28 | (0.03) | < .001 | 0.24 |  | 0.11 | (0.07) | .084 | 1.12 |
|  | *R*^2^ | 0.62 | | | |  | 0.61 | | | |  | 0.33 | | | |  | 0.44 | | | |

Note. Table presents unstandardized regression coefficients, their standard errors, standardized regression coefficients, and odds ratios (OR) for the categorical outcome. Bold font is used to highlight the effect significant in the parent-reported model, but not the self-reported victimization model. Peer victimization, teacher support, SES and school engagement were defined as latent variables. In self-reported model, N = 15103. In parent-reported model, N = 15104.

### Multilevel Analysis Exploring Schools Level Variables

In all the analyses presented in the manuscript, we used a complex-type analysis, in which we used clustering (each cluster representing a different school as the primary sampling unit) and stratification (non-deprived versus deprived areas). While we do not have a specific hypothesis regarding school-level variables in this paper, given that each school provides a unique social system, we also explored whether our predictor variables (teacher support and peer-victimization) yield a statistically significant effect at the school level. While the LSYPE study recruited participants from 659 schools, each represented by between 1 and 48 adolescents per school, we do not have any additional information about the schools (such as their ethnic composition), and some schools are represented by a very small number of pupils. Therefore, while we agree that schools create unique a social system, LSYPE dataset does not provide the optimum structure for a multilevel analysis.

Due to its complexity, this analysis required some adjustment in model specification, as using MLR estimator with Monte Carlo integration was too computationally demanding. The model was therefore defined using WLSMV estimator, and we changed the analysis type from complex to basic two-level. The model, presented in Table 5s, had a good fit, RMSEA = .04, CFI = .86, and despite using a different estimator compared to the main paper, in which we use MLR estimation, it overall replicated our findings at the within (individual) level. The notable exception was the effect of minority ethnic background on the probability of university enrolment, where using the WLSM estimator, we found a statistically significant negative effect of Black ethnicity on the probability of university enrolment (this negative direct effect did not reach a statistical significance using MLR estimator, *p* = .054), and the direct positive effect of Asian ethnicity was no longer significant (in the MLR model, this effect was statistically significant at *p* < .001). However, it should be noted that WLSMV uses probit coefficients for categorical outcomes, and MLR estimator uses logistic regression coefficients, which may explain these differences.

At the school-level, we found a statistically significant positive effect of teacher support on school engagement at T1, providing support to our findings that teacher support is associated with higher engagement in secondary school. While we could not test for the indirect effects in two-level model, given that all serial mediators had a significant effect on the following academic outcomes, the results suggest that the higher engagement will have a “knock-on” effect on academic outcomes in the following years. To interpret, schools in which pupils perceive their teachers as supportive have more engaged pupils in the early years of secondary school. We did not replicate the individual level effect of peer victimization, suggesting that schools with higher levels of peer victimization may not have overall less engaged pupils.

| Individual level variables | | | | | | | | | | | | | | | | | | | | | | | |
| --- | --- | --- | --- | --- | --- | --- | --- | --- | --- | --- | --- | --- | --- | --- | --- | --- | --- | --- | --- | --- | --- | --- | --- |
|  | *Dependent variable:* | T1 School engagement | | | |  | | T2 School engagement | | | |  | T3 University aspirations | | | |  | | T4 University enrolment | | | |  |
|  |  | *b* | (*SE*) | *p* |  | | *b* | | (*SE*) | *p* | |  | *b* | (*SE*) | *p* |  | | probit *b* | | (*SE*) | *p* |  |  |
| *Main effects:* | Peer-victimization | -0.02 | (0.00) | <.001 |  | | -0.01 | | (0.01) | .092 | |  | 0.02 | (0.01) | .104 |  | | -0.06 | | (0.02) | .013 |  |  |
|  | Teacher support | 0.55 | (0.01) | < .001 |  | | -0.03 | | (0.01) | .016 | |  | -0.05 | (0.03) | .045 |  | | 0.25 | | (0.04) | < .001 |  |  |
|  | Asian ethnicity | 0.22 | (0.01) | < .001 |  | | 0.05 | | (0.01) | < .001 | |  | 0.50 | (0.04) | < .001 |  | | 0.04 | | (0.06) | .529 |  |  |
|  | Black ethnicity | 0.13 | (0.01) | < .001 |  | | -0.02 | | (0.02) | .275 | |  | 0.50 | (0.04) | < .001 |  | | -0.26 | | (0.08) | .002 |  |  |
| *Mediators (serial):* | T1 School engagement |  |  |  |  | | 0.80 | | (0.02) | < .001 | |  |  |  |  |  | |  | |  |  |  |  |
|  | T2 School engagement |  |  |  |  | |  | |  |  | |  | 0.85 | (0.03) | < .001 |  | |  | |  |  |  |  |
|  | University aspirations |  |  |  |  | |  | |  |  | |  |  |  |  |  | | 0.63 | | (0.02) | < .001 |  |  |
| *Controls* | Household SES | 0.03 | (0.00) | < .001 |  | | 0.01 | | (0.00) | < .001 | |  | 0.18 | (0.01) | < .001 |  | | 0.17 | | (0.01) | < .001 |  |  |
|  | Gender | 0.05 | (0.01) | < .001 |  | | 0.01 | | (0.01) | .510 | |  | 0.27 | (0.02) | < .001 |  | | 0.05 | | (0.04) | .200 |  |  |
|  | *R*^2^ | 0.62 | | | |  | | 0.63 | | | |  | 0.32 | | | |  | | 0.42 | | | |  |
| School level variables | | | | | | | | | | | | | | | | | | | | | | | |
|  | *Dependent variable:* | T1 School engagement | | | |  | | T2 School engagement | | | |  | T3 University aspirations | | | |  | | T4 University enrolment | | | |  |
|  |  | *b* | (*SE*) | *p* |  | | *b* | | (*SE*) | | *p* |  | *b* | (*SE*) | *p* |  | | probit *b* | | (*SE*) | *p* |  |  |
| *Main effects:* | Peer-victimization | -0.15 | (0.08) | .060 |  | | -0.03 | | (0.08) | | .708 |  | -0.68 | (0.37) | .070 |  | | -0.74 | | (0.46) | .104 |  |  |
|  | Teacher support | 0.48 | (0.09) | < .001 |  | | 0.18 | | (0.11) | | .097 |  | -1.27 | (0.53) | .017 |  | | -0.20 | | (0.40) | .616 |  |  |
| *Mediators (serial):* | T1 School engagement |  |  |  |  | | 0.72 | | (0.16) | | < .001 |  |  |  |  |  | |  | |  |  |  |  |
|  | T2 School engagement |  |  |  |  | |  | |  | |  |  | 3.04 | (0.64) | < .001 |  | |  | |  |  |  |  |
|  | University aspirations |  |  |  |  | |  | |  | |  |  |  |  |  |  | | 1.19 | | (0.11) | < .001 |  |  |
|  | *R*^2^ | 0.61 | | | |  | | 0.77 | | | |  | 0.52 | | | |  | | 0.79 | | | |  |

Table 5s

A multilevel model reporting unstandardized regression coefficients (standard error in parentheses) at individual and school levels
